# PhosphoLipid transfer protein (PLTP) exerts a direct pro-inflammatory effect on rheumatoid arthritis (RA) fibroblasts-like-synoviocytes (FLS) independently of its lipid transfer activity

**DOI:** 10.1371/journal.pone.0193815

**Published:** 2018-03-22

**Authors:** Rachel Audo, Valérie Deckert, Claire I. Daien, Hélène Che, Jamila Elhmioui, Stéphanie Lemaire, Jean-Paul Pais de Barros, Catherine Desrumaux, Bernard Combe, Michael Hahne, Laurent Lagrost, Jacques Morel

**Affiliations:** 1 Department of Rheumatology, Montpellier University and Lapeyronie Teaching Hospital, Montpellier, France; 2 Montpellier University, Montpellier, France; 3 Institut de Génétique Moléculaire de Montpellier (IGMM), CNRS, UMR5535, Montpellier, France; 4 LNC Lipids, Nutrition and Cancer, INSERM UMR1231, Dijon, France; 5 University Bourgogne Franche-Comté, Dijon, France; 6 LipSTIC LabEx, Fondation de Coopération Scientifique Bourgogne-Franche Comté, Dijon, France; 7 University Hospital of Dijon, Dijon, France; 8 INSERM U1198, (MMDN), EiAlz Team, University Montpellier 2, EPHE, Montpellier, France; Macau University of Science and Technology, MACAO

## Abstract

Rheumatoid arthritis (RA) is a chronic inflammatory rheumatic disease with modification of lipids profile and an increased risk of cardiovascular events related to inflammation. Plasma phospholipid transfer protein (PLTP) exerts a lipid transfer activity through its active form. PLTP can also bind to receptors such as ATP-binding cassette transporter A1 (ABCA1). In addition to its role in lipoprotein metabolism and atherosclerosis, the latest advances came in support of a complex role of PLTP in the regulation of the inflammatory response, both with pro-inflammatory or anti-inflammatory properties. The aim of the present study was to decipher the role of PLTP in joint inflammation and to assess its relevance in the context of RA. PLTP expression was examined by western-blot and by immunochemistry. ABCA1 expression was analyzed by flow cytometry. Lipid transfer activity of PLTP and pro-inflammatory cytokines were measured in sera and synovial fluid (SF) from RA patients and controls (healthy subjects or osteoarthritis patients [OA]). FLS were treated with both lipid-transfer active form and inactive form of recombinant human PLTP. IL-8, IL-6, VEGF and MMP3 produced by FLS were assessed by ELISA, and proliferation by measuring ^3^H-Thymidine incorporation. RA synovial tissues showed higher PLTP staining than OA and PLTP protein levels were also significantly higher in RA-FLS. In addition, RA, unlike OA patients, displayed elevated levels of PLTP activity in SF, which correlated with pro-inflammatory cytokines. Both lipid-transfer active and inactive forms of PLTP significantly increased the production of cytokines and proliferation of FLS. ABCA1 was expressed on RAFLS and PLTP activated STAT3 pathway. To conclude, PLTP is highly expressed in the joints of RA patients and may directly trigger inflammation and FLS proliferation, independently of its lipid transfer activity. These results suggest a pro-inflammatory role for PLTP in RA.

## Introduction

Rheumatoid arthritis (RA) is an autoimmune disease characterized by chronic inflammation of joints leading to a progressive and irreversible joints destruction. The aggressive front of synovial tissue, called pannus, invades and destroys local articular structure. The pannus is characterized by neo-vessel formation mediated by angiogenic factors and a synovial hyperplasia, mainly composed of fibroblast-like-synoviocytes (FLS), combined with a massive infiltration of lymphocytes and macrophages. Both increased proliferation and insufficient apoptosis contribute to the local expansion of RA-FLS, which display pseudo-tumoral characteristics and directly participate in inflammation and joints destruction, through production of inflammatory mediators and metalloproteinase (MMP).

In later stages of RA, severe systemic complications arise with the main cause of mortality being cardiovascular disease (CVD). Indeed, in RA, there is an increased risk of CVD, correlated with markers of inflammation—C-reactive protein (CRP), erythrocyte sedimentation rate (ESR)—regardless of the usual CVD risk factors [1]. There is increasing evidence supporting an important link between chronic inflammation and CVD risk, particularly associated with endothelial dysfunction and early atherosclerosis onset [1]. Inflammation is also associated with variation of the lipid profile. In RA, inflammation is associated with a paradoxical inversion of the usual relationship between CVD risk and lipid levels [2]. Most studies showed that before any treatment, RA patients have lower levels of low density lipoproteins (LDL) and high density lipoproteins (HDL) cholesterol [3]. Total cholesterol and HDL cholesterol are inversely correlated with CRP. Furthermore, there is also qualitative change in lipoproteins, as oxidized LDL, small and dense LDL, as well as pro-inflammatory HDL are increased in RA [4, 5].

Liver X receptors (LXRs) are nuclear receptors activated by oxysterols (natural oxidative products of cholesterol), that are key modulators of lipid metabolism and transport. LXRs could also be involved in inflammatory diseases [6]. It has recently been shown that the LXRs pathway is the most up-regulated pathway in RA synovial fluid (SF) macrophages when compared to blood monocytes [7]. Furthermore, activation of LXRs, by ligands present within SF, augments TLR-driven cytokine secretion. Since the natural agonists of LXRs arise from cholesterol, and that cholesterol is increased in SF [8, 9], this provides a novel mechanism that can promote RA synovitis.

Phospholipid transfer protein (PLTP) gene is one of the LXRs targets and was also found to be overexpressed at the mRNA level in SF macrophages [7]. Like CETP (cholesteryl ester transfer protein), LBP (lipopolysaccharide-binding protein) and BPI (bactericidal/permeability-increasing protein), PLTP is also a member of the lipid transfer / LPS-binding protein (LT/LBP) gene family. PLTP is a ubiquitous, and multifaceted protein that can bind to and transfer a number of amphipathic molecules, including phospholipids, unesterified cholesterol, tocopherols, diacylglycerides and lipopolysaccharides (LPS). PLTP is therefore implicated in lipid and phospholipid transport in the bloodstream but also in HDL metabolism and remodeling (formation of β-HDL and large HDL) [10]. It also modulates the anti-inflammatory HDL property, impairing their ability to neutralize oxidized lipids, triggering atherosclerosis lesions [11]. PLTP-deficiency in mice is associated with a decreased susceptibility to atherosclerosis despite decreased HDL [12–14]. This atherogenic potential was also confirmed using PLTP transgenic rabbits [15].

Beyond its impact on lipoprotein metabolism, PLTP has recently been reported to modulate inflammation and immune responses. PLTP-deficient mice have less inflammatory manifestations, such as lower circulating levels of interleukin-6 (IL-6) [16, 17]. Reduced expression of IL-6 and infiltrating macrophages in aortic tissue of PLTP^-/-^ mice in comparison to the wild-type was recently reported in an experimental model of abdominal aortic aneurysm [18]. More recently, Desrumaux *et al*. demonstrated in PLTP-deficient mice a shift of T *helper* (Th) lymphocytes towards the anti-inflammatory subset Th2 [19]. However, other studies, mostly using a model of LPS-induced inflammation, suggest an anti-inflammatory role of PLTP [20–22]. Indeed, mortality increased after LPS injection in PLTP-KO mice [20]. Decrease in PLTP expression or activity was also shown to enhance the inflammatory responses in LPS and cigarette smoke exposition [21]. These anti-inflammatory functions could be explained by its capacity to bind and neutralize LPS, thereby reducing activation of innate immune system [20]. Yu *et al*. recently confirmed that PLTP was an essential acute-phase protein to suppress LPS-induced inflammation. It appeared to be dependent on its lipid binding capacities as only active PLTP could bind to LPS and form low cell toxic complexes *in vitro* [23]. In addition, PLTP could also have direct anti-inflammatory properties in macrophages through direct interaction with the ATP-binding cassette transporter A1 (ABCA1) and subsequent activation of the JAK2/STAT3 pathway [22].

Overall, and in light of earlier studies reporting potentiation by LXRs agonism of macrophage inflammatory response induced by LPS-mediated activation of TLR4 [7, 24], it remains unclear whether PLTP behaves as part of a pro- or anti-inflammatory response and may be dependent on the presence or absence of LPS in the vicinity of phagocytes.

Given that the LXRs pathway is upregulated in RA synovial tissue and that RA is characterized by inflammation, changes in lipid profiles and increased CVD, we investigated the expression and the role of PLTP in RA, focusing on FLS response. We focused on distinguishing between the direct pro-inflammatory and the indirect/LPS-dependent anti-inflammatory effect of PLTP, and establishing its pathophysiological relevance in the control of inflammatory response of FLS from RA patients.

## Materials and methods

### Subjects

All patient samples were collected in the Department of Rheumatology (Teaching Hospital of Montpellier). RA diagnosis was done according to the revised criteria of the 2010 American College of Rheumatology (ACR)/European League Against Rheumatism (EULAR) [25]. Synovial fluids were obtained from 23 RA patients, 19 patients with other inflammatory rheumatisms (including psoriatic arthritis [n = 3], other spondyloarthritis [n = 4], juvenile idiopathic arthritis [n = 2], chondrocalcinosis [n = 2] and unclassified inflammatory rheumatism [n = 8]) and 15 patients with osteoarthritis (OA) to compare their phospholipid transfer activity (PLTP activity). SF were centrifuged to remove cells (400*g*, 10 minutes) and treated with hyaluronidase (20 units/ml) for 30 minutes at 37°C, followed by centrifugation (10,000*g*, 5 minutes) to reduce viscosity of the sample. Sera from 10 healthy controls matched for age and gender with 10 RA patients were also included in the study. Clinical parameters (Disease index DAS28, C-Reactive Protein, erythrocytes sedimentation rate, and auto-antibodies (Rheumatoid factors, anti Cyclic citrullinated protein)) of the RA patients, assessed within 3 months of samples collection, are presented in Table 1. All donors gave written informed consent to participate in the study as approved by the Medical Ethics Committee of Nimes hospital, France [N°2012-A00592-41].

**Table 1:** Characteristics of patients included in this study (mean+/-SD).

	Synovial Fluid			Serum	
	RA	OIR	OA	HC	RA
N	23	19	15	9	10
Age (years) (+/- SE)	58 +/- 10	47 +/- 22	59 +/- 17	54±17	57±13
Sexe (%female)	65%	50%	36%	60%	70%
CRP (UI/ml) (+/- SD)	19.9+/-26.1	ND	ND	ND	ND
ESR (mm) (+/- SD)	20.2+/-20.1	ND	ND	ND	ND
RF+ (%)	67%	ND	ND	ND	ND
anti-CCP+ (%)	63%	ND	ND	ND	ND
erosion (%)	67%	ND	ND	ND	ND
DAS 28 (+/- SD)	3.4 +/-1,1	NA	NA	NA	ND

RA: rheumatoid arthritis, OIR: Other inflammatory Arthritis, OA: osteo-arthritis, HC: Healthy controls, CRP: C-Reactive Protein, ESR: Erythrocytes Sedimentation Rate, RF: Rheumatoid Factor, CCP: Cyclic citrullinated protein, DAS 28: Disease Activity Score 28, NA: Not Applicable; ND: Not Determined

### Determination of PLTP activity

PLTP activity was measured in serum and synovial fluid samples using a commercially available fluorescence activity assay (Roar Biomedical, New York, NY, USA) according to the manufacturer’s instructions. This fluorimetric assay measures the transfer (unquenching) of fluorescent phospholipid from donor to acceptor synthetic liposomes. Phospholipid transfer rates were calculated using the initial slope of the phospholipid transfer curve and were expressed as initial phospholipid transfer rate (i.e., increase in fluorescence arbitrary units (AU) per minute).

### Inflammatory parameter measurements

Concentrations of IL-6, IL-1β and TNF-α in synovial fluids were quantified using a Milliplex MAP Human Cytokine/Chemokine Magnetic Bead Panel kit (Millipore, Billerica, MA). The assays were performed according to the manufacturer’s instructions. Standards and samples were analyzed on a LuminexR^®^ apparatus (Bio-Plex 200, BioRad, München, Germany) using the BioPlex Manager Software (Version 5, BioRad, Hercules, CA).

Net mass concentrations of LPS were measured in synovial fluids through the direct quantitation of 3-hydroxmyristate by liquid chromatography–mass spectrometry (LC-MS) according to the general procedure previously described [26].

### Preparation and treatment of FLS

FLS were isolated as described previously [27], from synovium obtained from OA patients and RA patients meeting the American College of Rheumatology criteria for RA (ACR-EULAR 2010). Briefly, fresh synovial tissues were broken down and digested in a solution of dispase (2,4 mg/ml) (Gibco, Cergy Pontoise, France), collagenase (250 U/ml) (Sigma) and DNAse (10000 U/ml) (Calbiochem, Fontenay sous Bois, France). Synovial fibroblasts were cultured in RPMI-1640 supplemented with 10% fetal calf serum (FCS) at 37°C, in a humidified atmosphere with 5% CO_2_. Cells were used at passage 4–10, when they comprise a homogeneous population of fibroblasts. Upon reaching confluence, the cells were passaged by brief trypsinization. For experimentation, the FCS in the RPMI media was progressively decreased from 10% to 1%, with final starvation for 12 to 24 hours in RPMI-media containing 1% FCS [27].

To test the effect of recombinant human PLTP (rhPLTP) on FLS cytokines production, FLS were seeded in 12-well flat-bottom culture plates at a density of 1x10^5^ cells/well. Cells were cultured in RPMI with decreasing concentration of FCS (10 and 5%) and for 12 hours with RPMI 1% FCS before stimulation. FLS were then stimulated 24 hours with rhPLTP at indicated concentrations.

To evaluate the effect of rhPLTP on FLS proliferation, FLS were seeded in 96-well flat-bottom culture plates at a density of 1x10^4^ cells/well. Cells were cultured in RPMI with decreasing concentration of FCS (10 and 5%) and then synchronized for 24 hours with RPMI 1% FCS before stimulation. FLS were then stimulated with rhPLTP at indicated concentrations, TNF-α (20ng/ml; R&DSystem) or IL-1β (10 ng/ml, Miltenyi Biotech) for 48 hours. Each condition was tested in quadruplicate. In order to test specificity, either rhPLTP was pre-incubated for 30 min with an anti-PLTP antibody (10μg/ml; H00005360-M01, Abnova) before addition to FLS, or FLS were pre-incubated for 30 min with glyburide (25 μM, Invitrogen), a chemical inhibitor of ABCA-1 before addition of rhPLTP. In order to test an eventual contamination of the rhPLTP by endotoxin, rhPLTP preparation was pre-incubated with polymyxin B for 15 min at 37°C and then added to the cells.

### Preparation of recombinant human PLTP

RhPLTP was extracted from milk of PLTP transgenic rabbit females, which were generated according to the general procedure as previously described [28–30]. In active preparations of rhPLTP, phospholipid transfer activity was equivalent to that measured in normolipidemic human serum and the mean concentration of PLTP was around 4 μg/ml [31]. RhPLTP preparations were 0.22μm filter-sterilized and diluted in RPMI 1% SVF at 2 μg/ml, 1μg/ml or 0.5 μg/ml. Inactivation of lipid transfer activity of PLTP was obtained by heating rhPLTP preparations for 2 hours at 65°C. The total loss of phospholipid transfer activity was checked by using the fluorescence activity assay as described above.

### Cell proliferation assay

FLS proliferation was evaluated by measuring DNA synthesis assessed by incorporation of tritiated [^3^H] thymidine. FLS were stimulated for 48 hours and then pulsed with [^3^H] thymidine (1 Ci/ well) for 24 hours. FLS were then lysed using a round of freeze-thaw cycles and transferred onto a membrane filter using Cell Harvester 12 (Molecular Devices, Wokingham, UK). [^3^H] thymidine incorporated into DNA was quantified using a scintillation counter TriCarb 20800 TR (PerkinElmer, Massachusetts USA). Results are presented as stimulation index (arithmetic mean of cpm from quadruplicate of stimulated culture / arithmetic mean of cpm from quadruplicate of non-stimulated culture).

### Il-6, IL-8, VEGF and MMP3 measurement in FLS culture supernatants

IL-6, IL-8, VEGF and MMP3 were assayed in FLS culture supernatants using commercially enzyme-linked immunosorbent assay (ELISA) kits (900-K16; 900-K18; 900-K10, from Peprotech and DMP300 from R&D Systems, respectively).

### Flow cytometric analysis

RA FLS were harvested with 1 nM EDTA and suspended in PBS supplemented with 2% SVF. Fifty to hundred thousand cells were incubated for 20 minutes on ice in with 0.5 mg of mouse anti-ABCA1 antibody (from R&D Systems). As a negative control, cells were incubated with isotype control antibody. Cells were then washed in FACS Buffer and subsequently incubated for 20 minutes on ice with a PE-conjugated anti-mouse antibody (Pharmingen). Viable cells were selected by TOPRO-3 exclusion and analysis performed using FACSCanto II (Becton Dickinson). To assess intracellular expression, FLS were permeabilized with Cytofix/Cytoperm buffer for 30 min on ice (BD Biosciences, Le pont-de-Claix, France) and 1X Perm/wash Buffer (BD Biosciences) 10 minutes on ice before staining.

### Immunohistochemical experiments

Synovial tissues were isolated from patients who were undergoing total joint replacement surgery or synovectomy. Briefly, tissues were fixed in formalin and embedded in paraffin. Immunochemistry was performed after deparaffinization, rehydratation, and unmasking by heating in 10 mmol/L sodium citrate buffer (pH 6). Unspecific sites were saturated with PBS containing 10% horse serum and endogenous peroxidase activity was blocked using 3% hydrogen peroxide. Sections were then incubated with a mouse monoclonal antibody to PLTP (5μg/ml; ab57273; Abcam) or mouse anti-CD68 antibody (2.5 μg/ml; M0814; Dako). HRP-coupled secondary antibodies were added and incubated at room temperature for 1 hour. Visualization of immunohistochemical section was performed using Nanozoomer slide scanner (Hamamatsu Photonics). Double staining using antibodies specific for macrophages (mouse anti-CD68 antibody (2.5 μg/ml; M0814; Dako) or RA-FLS (mouse anti-alpha-smooth muscle Actin (α-SMA) (1 μg/ml; 1A4; eBioscience) and PLTP (rabbit anti-PLTP antibody (1/200- NB400-106; Novus Biological) was also performed. As a negative control, sections were incubated with isotype control antibodies (mouse IgG MOPC21 (M7894-Sigma) or rabbit IgG (X0936-Jackson Immunoresearch). Visualization of immunofluorescence in synovial section was performed using Leica DM 6000 microscope (Leica Microsytems).

### Western blot

For PLTP expression comparison in FLS from RA and OA patients, all primary cultures were used at passage 4 [32]. Proteins were isolated from primary culture as previously described [33]. Protein fractions were stored at -80°C until WB experiments. Before immunoblot experiments, protein concentrations were determined using the standard procedure for microplate assays using the MicroBCA^™^Protein assay Reagent Kit (Pierce, Cat.-No 23235) as recommended by the manufacturer. An equal amount of cell lysate (30μg) was mixed with 0.3 volume of 4 x sample buffer containing DTT (10 mM) and resolved by 10% polyacrylamide gel.

For signaling experiments, FLSs were stimulated with PLTP at the indicated concentrations and the reaction was stopped by plunging into ice. Cells were washed two times in ice-cold PBS buffer (Invitrogen Saint Aubin, France) and lysis buffer (50 mM Tris, pH 6.8; 2% glycerol; 2% SDS and 1% glycerol, β-mercaptoethanol and 0.001% Bromophenol blue) was added directly to the cells for 5 min followed by cell scratching. An equal amount of cell lysates was resolved by 10% polyacrylamide gel, and immunoblot analysis was performed.

An ECL plus detection system (RNP2232; GE Healthcare) was used to detect specific protein bands. Blots were developed, scanned and densitometric signals were determined using the NIH image J 1.63 software. PLTP was normalized to β-actin levels. Expression level of PLTP was expressed as a ratio relative to the mean expression of PLTP in all FLS tested (RAFLS and OAFLS). To detect PLTP, mouse monoclonal antibodies to PLTP (2μg/ml; ab57273; Abcam) or anti- β-actin (1/10,000; A5441; Sigma) and horseradish peroxidase-conjugated rabbit anti-mouse IgG (1:10,000; P0260; Dako) were used. Antibodies used to detect STAT3 were purchased from Cell Signalling (anti-phospho-STAT3 (Tyr705), clone D3A7; 1/1000 #9145 or anti-STAT3, clone D1A5; 1/1000 #8768).

### Statistical analysis

Results were expressed as means ± standard deviations (SD). Mann-Whitney U test (non-parametric) for unpaired set of data and Wilcoxon matched pairs test for paired set of data were used. Unless when indicated, two-tailed p values were calculated. Correlations were determined by Spearman correlation. All statistical analyses were calculated using GraphPad Prism version 4.00 (GraphPad Software, San Diego, CA).

## Results

### PLTP is expressed in RA joints

**Fig 1:**
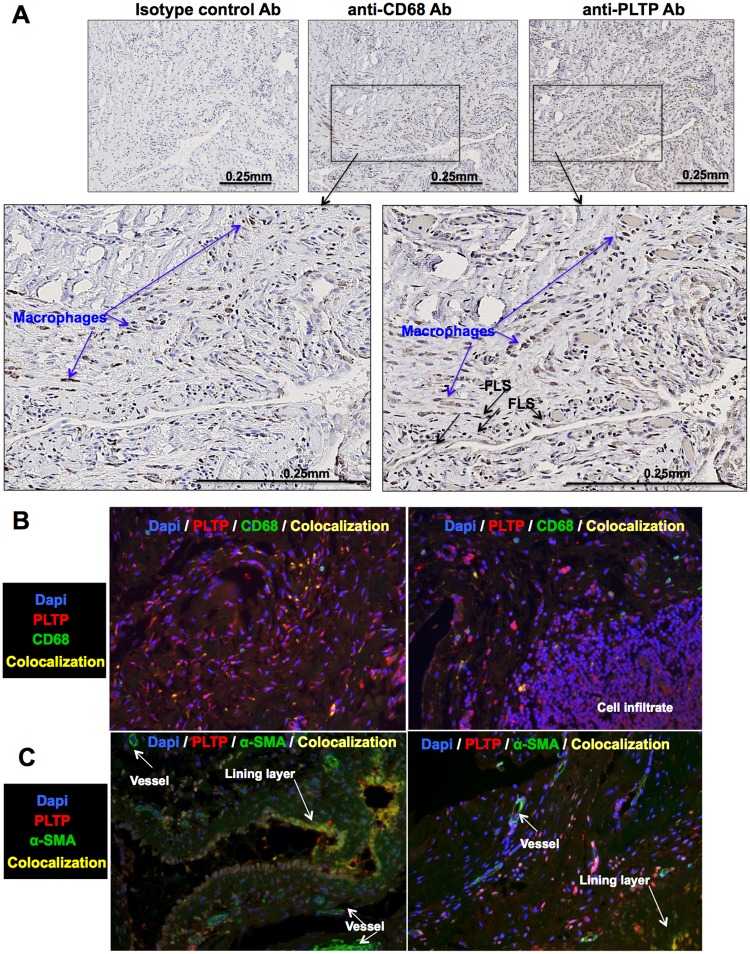
PLTP expression in RA synovial tissues. For immuno-histological analysis, (**A**) synovial tissue sections from RA patients (n = 5) were stained for PLTP or macrophages (CD68^+^ cells). Representative images obtained for immunohistological staining are shown. Blue arrows show macrophages (non-exhaustive), determined as CD68^+^ cells. FLS are determined with morphological features and CD68^+^ staining (black arrow; non-exhaustive). (**B, C**) Double staining was performed to visualize localization (n = 3) of PLTP with macrophages (CD68^+^) (B) or RA-FLS (α-SMA+) (C). Fluorescence was analyzed at 20x magnification. Overlay is shown to visualize co-localization of PLTP in macrophages (CD68^+^) (B) or RA-FLS (α-SMA+) (C) or PLTP expression in infiltrate. Original magnification: 20x. Separate images can be found in S1 Fig.

We first assessed the expression of PLTP within the joints of RA patients. PLTP expression was analyzed in the synovial tissues of RA patients by immunochemistry (n = 5). As shown in Fig 1A, PLTP can be detected in the synovial tissues, showing diffuse staining co-localized in the macrophage-rich area (arrows in Fig 1A) and but also in fibroblast-like-synoviocytes (FLS) (Fig 1A). Localization within macrophages and RA-FLS was confirmed by immunofluorescence (Fig 1B and S1 Fig). Indeed, PLTP colocalized with CD68+. In healthy synovium, alpha smooth muscle Actin (α-SMA) expression was only found in blood vessels. In contrast, in RA synovial tissue, α-SMA expression was also found in FLS, especially in the lining layer [34]. We therefore used this marker and found that α-SMA and PLTP co-localized, supporting co-localization of PLTP with RA-FLS (Fig 1C). In addition, PLTP staining was found in infiltrating immune cells (Fig 1B, right panel and S1 Fig).

**Fig 2:**
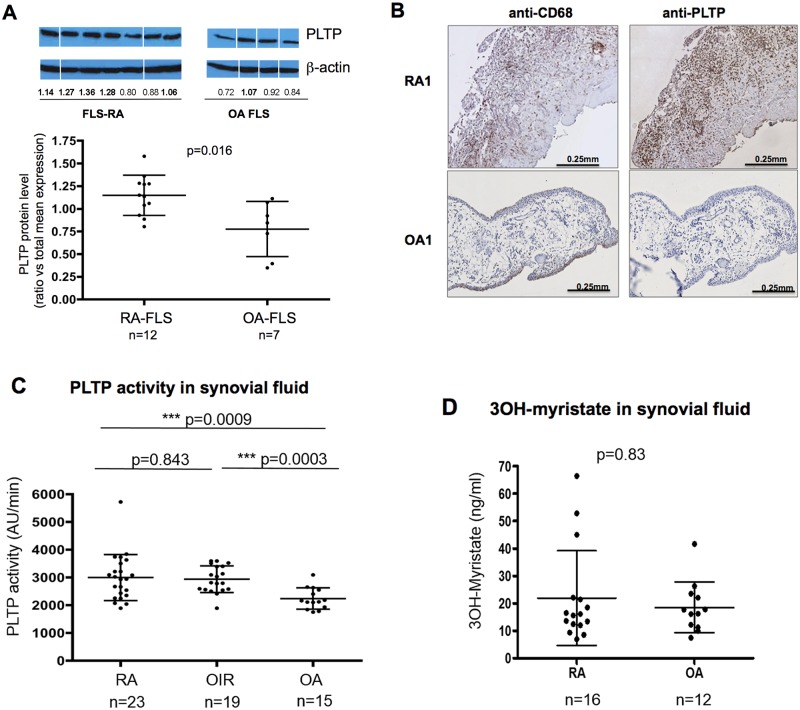
PLTP expression and activity in RA and OA joints and 3OH-myristate levels in synovial fluid of RA and OA patients. **(A**) PLTP protein level in FLS was quantified by Western blot analysis, normalized using β-actin and then expressed as a ratio vs mean expression level in all FLS tested. Representative images obtained from one gel are shown. (Protein extracts were migrated stained and exposed at the same time. Original Western blot can be found in S2 Fig). All data are shown as mean ± SD and statistical analysis performed using Mann-Whitney test. **(B)** Immunohistological analysis of PLTP expression in synovial tissue from 5 RA and 6 OA patients stained for PLTP and macrophages (CD68^+^ cells); All RA synovial tissue showed positive stainings while only 2 out 6 OA tissues were positive for PLTP staining. Representative stainings are shown. (**C**) Phospholipid transfer activity was measured in synovial fluid by fluorescence as described in Materials and Methods. Data are expressed as mean increase in fluorescence per minute (AU /min) and represented as mean ± SD. Statistical analyses were performed using Mann-Whitney test (**D)** Net mass concentration of LPS was assessed in SF of RA patients (n = 16) compared with osteoarthritis patients (OA) (n = 12) by the direct quantitation of lipid A 3-hydroxymyristate by liquid chromatography–mass spectrometry (LC-MS analysis). Statistical analyses were performed using Mann-Whitney test.

Western-blot analyses displayed significantly higher PLTP protein levels in RA-FLS than in OA-FLS (1.15±0.22 and 0.77±0.34 respectively i.e. 48% increased, p = 0.016) (Fig 2A). This was confirmed by immunohistochemistry analysis of tissue sections of joints from established RA patients displaying elevated PLTP expression in synovial tissue section when compared to those of OA patients (Fig 2B).

We also analyzed the level of PLTP activity in synovial fluids of patients with RA or other inflammatory rheumatisms (OIR) and osteoarthritis (OA). Characteristics of included patients are presented in Table 1. PLTP activity was significantly increased in SF of RA and OIR patients (2953±849 AU/min and 2930±481 AU/ min, respectively) compared to OA patients (2180±355 AU/min) (Fig 2C).

Because PLTP was suggested to reduce LPS-mediated inflammation and to have a role in endotoxemia [20], we measured LPS amount (or endotoxin) in SF by quantifying its component lipid A 3-hydroxymyristate as previously described [26]. LPS amount detected in SF from RA and OA patients did not differ significantly (Fig 2D).

Interestingly, PLTP activity measured in SF from RA patients was significantly higher than PLTP activity measured in serum samples with mean values of 2953±849 and 1713 ± 355 AU/min, respectively (p<0.0001). No difference could be detected between serum PLTP activity in RA patients and healthy controls (1713±355 and 1472±392 AU/min respectively, p = 0.43).

### PLTP activity in synovial fluid correlates with levels of pro-inflammatory cytokines

Pro-inflammatory cytokines (IL-1β, TNF-α and IL-6) were also measured in SF of RA and OA patients. IL-6 and TNF-α concentrations were significantly increased (p = 0.018 and p = 0.03, respectively) in SF from RA patients (n = 23) compared to OA patients (n = 13) (S3 Fig). Though not significant, the same tendency was also observed for IL-1β (p = 0.15) (S3 Fig). In patients with RA, PLTP activity correlated with IL-1β, and IL-6 but surprisingly not with TNF-α, while no correlation could be established in OA patients (Table 2).

**Table 2:** Correlation between PLTP activity and pro-inflammatory cytokines in synovial fluids. (Spearman correlation test).

	RA (n = 23)		OA (n = 13)	
	r	p	r	p
TNF-α	0.21	0.31	0.23	0.40
IL-1β	0.53	**0.0088**	0.18	0.54
IL6	0.53	**0.0086**	0.21	0.43

RA: rheumatoid arthritis; OA: osteoarthritis.

### PLTP stimulates cytokine production in FLS and proliferation independently of its lipid transfer ability

**Fig 3:**
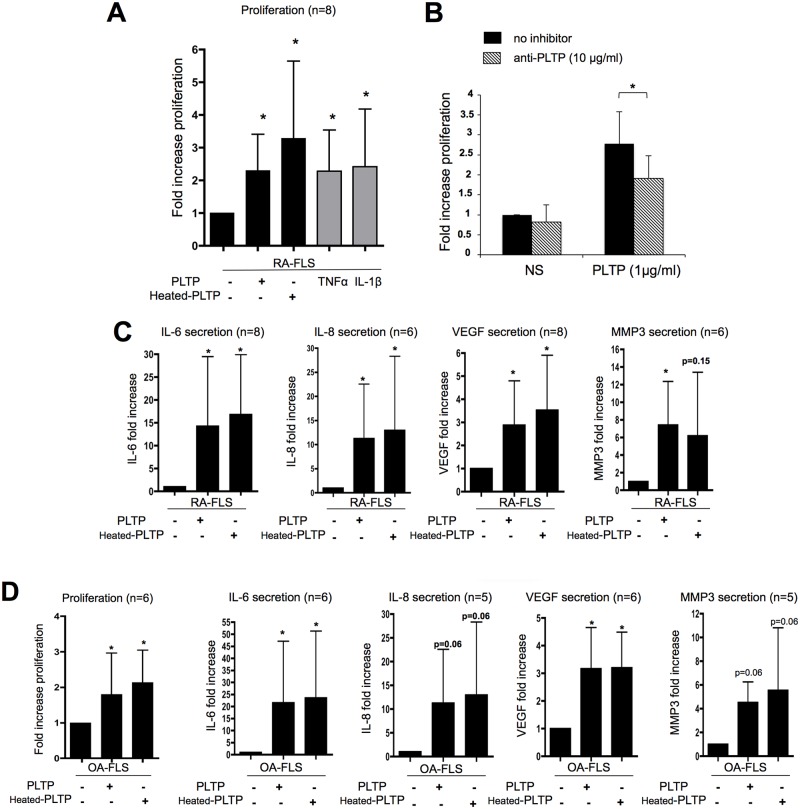
Recombinant PLTP induced FLS proliferation and cytokine production independently of its lipid transfer ability. **(A)** RA-FLS were stimulated for 48 hours with 2μg/ml of PLTP or heat inactivated-PLTP (heated-PLTP) and proliferation was evaluated using [^3^H] thymidine incorporation during the last day of stimulation. Results are expressed as mean fold increase ± SD (n = 8). Statistical differences were assessed by Wilcoxon matched paired test. *p < 0.05 versus unstimulated conditions; TNF-α and IL-1β: positive controls of proliferation. (**B**) Blockade of PLTP decreased the effect of rhPLTP on FLS proliferation. Cells were treated with PLTP pre-incubated or not with anti-PLTP antibody (n = 5). *p< 0.05 (one-tailed p value), Wilcoxon matched paired test. (**C)** Effect of PLTP on RA-FLS cytokine production. FLS were stimulated for 24 hours with PLTP or heated-PLTP (2μg/ml). Supernatants were then collected and assessed for cytokines (IL-6, IL-8, VEGF and MMP3) production by ELISA. Results are expressed as mean fold increase ± SD. Statistical differences were assessed by Wilcoxon matched paired test. *p< 0.05 versus unstimulated condition (n = 6 to 8).(**D**) OA-FLS were treated with either native PLTP or heated-PLTP and analyzed for proliferation (left panel) and cytokine production (right panels) as previously described (n = 5 or 6; *p< 0.05 versus unstimulated condition). RA-FLS and OA-FLS responses were compared using Mann Whitney test and no significant differences could be demonstrated.

We tested the effect of native rhPLTP and heat-inactivated rhPLTP on FLS proliferation and inflammatory response. Native PLTP retains its lipid transfer activity, while heated PLTP is inactive. The purpose of comparing rhPLTP and heated rhPLTP action was to distinguish between lipid transfer activity-dependent and -independent functions of the protein. In the first set of experiments, FLS were either untreated or stimulated with TNF-α or IL-1 β (as positive control conditions) or stimulated with native and heat-inactivated rhPLTP (Fig 3A and S4 Fig). We observed that native rhPLTP induced a significant increase in RA-FLS proliferation (by 2.35 ± 1.1 at the highest PLTP concentration tested (2μg/ml), p<0.05, n = 8) (Fig 3A). Importantly, heat-inactivated rhPLTP was also able to significantly increase RAFLS proliferation (p<0.05, n = 8) (Fig 3A), suggesting that PLTP induced proliferation independently of its lipid transfer activity. Of note, no differential responses of FLS from RA patients or OA patients were observed, suggesting that both are able to respond to PLTP (Fig 3D, left panel).

In order to establish that the effect of rhPLTP was independent of endotoxin/LPS and to assess purity of the rhPLTP preparation, cells were treated with rhPLTP in the presence of polymixin B as an LPS scavenger. Polymixin B treatment did not modify the rhPLTP-mediated proliferation, confirming that the increase in inflammatory response was not due to a putative contamination of the rhPLTP preparation by LPS (https://figshare.com/s/c79464fec9c5a01ad056). To further assess the specificity of rhPLTP preparation on FLS responses, we evaluated the effect of PLTP in the presence of anti-PLTP antibody. Anti-PLTP antibody significantly decreased rhPLTP-induced proliferation (p = 0.031, n = 5; Wilcoxon matched paired test) (Fig 3B).

In another set of experiments, FLS were stimulated with native or heat-inactivated rhPLTP at indicated concentrations and inflammatory response (e.g. IL-6, IL-8, VEGF and MMP3 production) was assessed. Native rhPLTP as well as heat-inactivated rhPLTP significantly induced IL-6, IL-8, VEGF and MMP3 secretion by RA FLS (Fig 3C and S4 Fig). Of note, PLTP also stimulated OA-FLS cytokine production (Fig 3D).

### ABCA1 is expressed at the cell surface of RA-FLS and PLTP activates the JAK/STAT pathway

**Fig 4:**
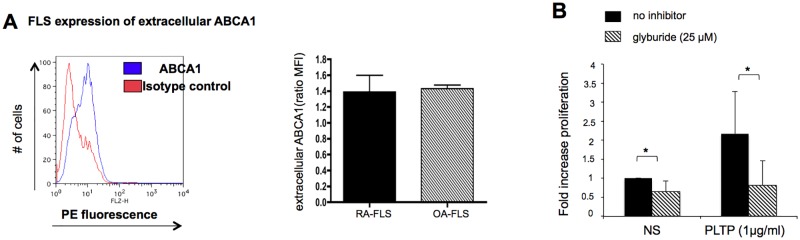
ABCA1 is expression in FLS and glyburide decreased the effect of rhPLTP on FLS proliferation. **(A)** Analysis of ABCA1 at the cell surface was performed by flow cytometry in FLS from 9 RA donors and 4 OA donors. The ratio of the mean fluorescence intensity (MFI) of ABCA1 staining versus isotype control antibody staining was calculated and shown as mean ± SD. **(B)** Glyburide decreased the effect of rhPLTP on FLS proliferation. Cells were pre-incubated with the ABCA1 inhibitor, glyburide and then stimulated for 48 hours with rhPLTP in the presence of glyburide. Proliferation was evaluated by thymidine incorporation. Results are presented as fold increase vs unstimulated conditions (no inhibitors and RPMI only) (n = 5, p<0.05 (one-tailed p value), Wilcoxon paired test).

Because the direct PLTP effect on macrophages was suggested to be mediated by ABCA1 [22], we investigated ABCA1 expression in RA-FLS by FACS analysis. ABCA1 is detected at the cell surface (Fig 4A). We compared ABCA-1 expression levels by FACS in FLS from RA and OA patients and both displayed a similar cell surface expression (Fig 4A)

We evaluated the effect of rhPLTP in the presence of glyburide, a chemical inhibitor of ABCA1. RhPLTP-induced proliferation of FLS was significantly decreased by glyburide (n = 5, p<0.05) (Fig 4B) and, surprisingly, glyburide also decreased basal proliferation of RAFLS. In contrast, glyburide did not alter TNF-α and IL-1β induced proliferation (https://figshare.com/s/3dfb2e48835c5dc25c14), suggesting that glyburide specifically inhibited PLTP-induced proliferation.

**Fig 5:**
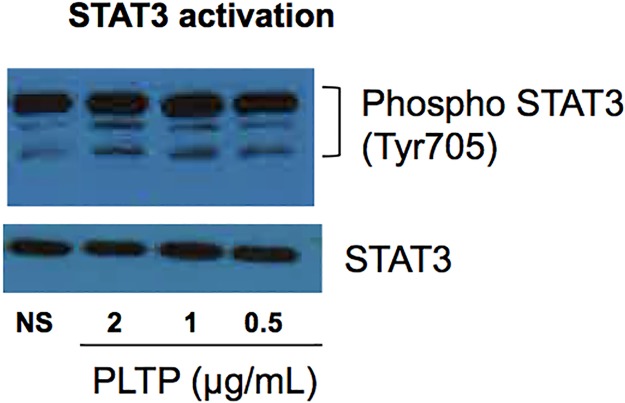
PLTP activated STAT3 pathway. RA-FLS were stimulated for 24 hours with rhPLTP at the indicated concentrations. Cell lysates were analyzed by Western blot for phosphorylation of STAT3 (Tyr705). Band intensities were normalized to the corresponding band intensities for STAT3. Representative Western blots are shown (n = 3).

PLTP was described to induce JAK/STAT pathway through ABCA1 binding [22, 35]. We therefore assessed the effect of PLTP treatment on STAT3 phosphorylation in RA-FLS. As shown in Fig 5, phosphorylation of STAT3 was induced by rhPLTP (Fig 5). Of note, both forms of rhPLTP were able to induce STAT3 phosphorylation demonstrating that other functions than lipid transfer activity are preserved after heating (S5 Fig).

## Discussion

In the present study, we demonstrated for the first time that PLTP is overexpressed in synovial tissue of patients with chronic inflammatory rheumatisms, such as RA, when compared to OA. In addition, we showed that RA but not OA patients displayed elevated levels of PLTP activity in synovial fluid, which were correlated with pro-inflammatory cytokine (IL1β, IL-6) levels. Consistent with these observations, we found that, *in vitro*, rhPLTP was able to induce FLS proliferation and production of cytokines (IL-8, IL-6, VEGF and MMP3). PLTP seems to exert its effects independently of its lipid transfer activity and through the STAT3 pathway. Thus, increased PLTP in the joints of RA patients is likely to play a role in RA pathogenesis.

In addition to its function in lipoprotein metabolism, PLTP belongs to the positive acute phase reactants family, with a potential role in inflammation and innate immunity. Indeed, PLTP activity was found elevated in sera from patients with acute inflammation and clinical severe sepsis [36–38]. Furthermore, an association between PLTP activity and inflammatory marker CRP was found in patients with CVD and type 2 diabetes [38–40]. In the present study, we reported that serum PLTP activity does not appear to be altered in RA patients compared to healthy controls. However, in RA patients, PLTP activity is significantly higher in SF than in serum, suggesting a local effect at the joints level rather than at the systemic level. PLTP activity levels in SF are significantly higher in chronic inflammatory rheumatisms (RA and OIR) than in OA. RA SF displayed elevated levels of PLTP activity, which correlated positively with pro-inflammatory cytokines. Thus, PLTP activity is related with RA. However, the relationship between inflammation and PLTP seems to be cytokine-specific. The correlations suggest that the relation between PLTP and inflammation is more dependent on IL-6 and IL-1β than on TNF-α. Asquith et al demonstrated that the LXRs pathway is the most up-regulated pathway in RA synovial macrophages, including PLTP overexpression at the mRNA level [7]. In the present study, we confirmed higher PLTP expression in RA synovial tissue at the protein level, which appears to originate mainly from macrophages since we detected PLTP in the macrophage-rich area by immunohistochemistry, but also from RA-FLS. Recent studies have suggested an association of microbial infections with RA initiation and perpetuation [41]. Because of the link between PLTP and endotoxin (LPS) neutralization, we tested the presence of LPS in RA synovial fluid. No significant amounts of LPS were measured in SF from RA patient compared to OA, suggesting that the higher activity of PLTP in RA synovial fluid was not related to the presence of endotoxin. In addition, because inflammation is associated with variation of the lipid profile, it can be speculated that the association between PLTP and inflammatory cytokines could be linked to the relationship between inflammation and lipids. However, we did not detect significant difference in lipid levels in SF from RA and OA patients (S3 Fig), suggesting a direct link between PLTP and inflammation.

Taken together, these results suggest that increased expression of PLTP in the joints of RA patients could have a role in RA pathogenesis. We therefore tested the effect of rhPLTP on FLS responses. We demonstrated that rhPLTP significantly increased FLS production of inflammatory cytokines (IL-6 and IL-8), VEGF (angiogenic mediator) and MMP3 (an important protease in joint damage). In addition, PLTP significantly increased FLS proliferation. Therefore, increased PLTP could participate in synovial hyperplasia, inflammation as well as in destruction of the joints. More importantly, both native and heat-inactivated rhPLTP were able to greatly increase proliferation of FLS and cytokine production. Heating probably led to conformational changes of PLTP that induce the loss of its phospholipid transfer activity whereas other functions of the protein are preserved. The purpose of comparing rhPLTP and heated rhPLTP action was to distinguish between dependent and independent lipid transfer activity of the protein. Thus, our data suggest that the pro-inflammatory properties of PLTP on FLS were not linked to its lipid transfer activity and they come in support of those reported by Vuletic et al [22] who, by using a PLTP mutant devoid of lipid transfer activity, have also provided evidence that PLTP-mediated lipid transfer is not required for its ability to modulate cell inflammatory processes. In addition to studies reporting that PLTP interacts with ABCA1 to enhance cholesterol efflux from cells and that PLTP/ABCA1 interaction activates JAK2 [35, 42], Vuletic and colleagues were the first to demonstrate that PLTP could modulate inflammatory process through binding to ABCA1 [22]. Indeed, glyburide, a chemical inhibitor of the ABCA1, and siRNA targeting ABCA1 counteracted PLTP-induced activation of the JAK2/STAT3 pathway [22]. In the present study, we showed that ABCA1 is expressed at the cell surface of RA-FLS and that glyburide decreased the effect of PLTP on FLS proliferation, suggesting that ABCA1 might mediate the pro-inflammatory effect of PLTP on these cells. In addition, we found that both forms of rhPLTP induce STAT3 activation in FLS. Therefore, it can be speculated that ABCA1 mediates the pro-inflammatory role of PLTP on RA-FLS through activation of the JAK2/STAT3 pathway. Activation of this pathway does not require the lipid transfer activity of the protein.

However, the pro-inflammatory potential of PLTP-mediated activation of STAT3 on RAFLS contrasts with the demonstration that PLTP could have an anti-inflammatory effect on macrophages [22]. In line with PLTP differential effect, STAT3 has a dual role in inflammation. As reported by Williams et al [43], STAT3 activity can suppress both IL-6 and TNF-α production in macrophages whereas, in synovial fibroblasts, STAT3 rather enhanced IL-6 production, suggesting that the cellular context plays an important role in dictating whether STAT3 drives a pro- or an anti-inflammatory response [43]. In addition in RA, STAT3 was shown to support inflammation through activation of FLS [44, 45] and differentiation of effector T cells [46]. Moreover, blocking of JAK/STAT pathways is a therapeutic approach for the treatment of inflammatory diseases, including RA [47–49]. Taken together, all these data come in support of a pro-inflammatory role of PLTP on RA FLS. PLTP-mediated activation of STAT3 pathway could therefore partially be partially responsible for the dual effect of PLTP in inflammatory responses.

Thus, depending on cell type, cell context, and localization (systemic vs cellular expression), PLTP could have a differential role in inflammatory responses. This could possibly explain why some studies suggested a protective role of PLTP, in atherosclerosis, for example when PLTP is expressed in macrophages [50][51] or in the context of endotoxemia [23]. Furthermore, not only the activity but also the total mass of PLTP should be considered, as our study confirmed a direct and significant effect of PLTP in inflammatory responses that is independent of its lipid transfer activity.

In the present study, we clearly demonstrated pro-inflammatory and proliferative effects of PLTP on FLS, independent of its lipid transfer activity. Increased PLTP in the joint of RA patients could therefore directly target FLS, and participate in inflammation, joint destruction and synovitis observed in RA. PLTP could therefore be an interesting therapeutic target to control RA synovitis.

## Supporting information

S1 Fig PLTP was expressed in synovial tissue from RA patients. A) Double staining was performed to visualize PLTP localization. Synovial tissue sections from RA patients were stained for PLTP and macrophages (CD68^+^, left panel), or PLTP and RA-FLS (α-SMA+ cells) (n = 3). Fluorescence was analyzed at 20x magnification. Overlay is shown to visualize colocalization of PLTP in macrophages or in RA-FLS. Representative images obtained for immunohistological staining are shown. Original magnification: 20x.
(TIF)

S2 Fig PLTP expression in FLS by Western blot. PLTP protein level in FLS was quantified by Western blot analysis, normalized using β-actin and then expressed as a ratio vs mean expression level in all FLS tested. A representative image is shown (RA: rheumatoid arthritis FLS, OA: osteoarthritis FLS; DF: normal dermal fibroblasts).
(TIF)

S3 Fig IL-6 and TNF-α are significantly increased in SF from RA patients (A) while lipids levels are not different (B). **(A)** Synovial fluids samples from patients were tested for IL-6, TNF-α and IL-1β concentrations using Milliplex MAP Human Cytokine/Chemokine Magnetic Bead Panel kit (Millipore, Billerica, MA). **(B)** Plasma lipids (total cholesterol, triglycerides and phospholipids) were assayed using commercially available kits on an Indiko Clinical chemistry analyzer (Thermo Fisher Scientific, Finland) according to the manufacturer’s instructions. Results are expressed as mean ± SD and statistical analysis performed using the Mann-Whitney test.
(TIF)

S4 Fig Recombinant PLTP dose-dependently induced FLS proliferation (A) and cytokine production (B). **(A)** FLS were stimulated for 48 hours with native PLTP at indicated concentrations and proliferation was assessed using [^3^H] thymidine incorporation during the last day of stimulation. Results are expressed as mean fold increase ± SD (n = 6). Statistical differences were assessed by Wilcoxon matched paired test. *p < 0.05 versus unstimulated conditions; NS: unstimulated **(B)** Effect of PLTP on FLS cytokine production. FLS were stimulated with native PLTP at indicated concentrations. Supernatants were then collected and assessed for cytokines (IL-6, IL-8, VEGF and MMP3) production by ELISA. Results are expressed as mean fold increase ± SD (n = 5 to 6). Statistical differences were assessed by Wilcoxon matched paired test. *p< 0.05 versus unstimulated conditions.
(TIF)

S5 Fig Both native rhPLTP and heat-inactivated rhPLTP activated STAT3 pathway. RA-FLS were stimulated for 24 hours with native PLTP (PLTP) or heat-inactivated PLTP (Heated PLTP) at the indicated concentrations. Cell lysates were analyzed by Western blot for phosphorylation of STAT3 (Tyr705). Band intensities were normalized to the corresponding band intensities for STAT3.
(TIF)
