# Supplementary figures and images for: PhosphoLipid transfer protein (PLTP) exerts a direct pro-inflammatory effect on rheumatoid arthritis (RA) fibroblasts-like-synoviocytes (FLS) independently of its lipid transfer activity
Source: PLoS One. 2018 Mar 22;13(3):e0193815. doi: 10.1371/journal.pone.0193815 (PMC5863966; doi:10.1371/journal.pone.0193815)

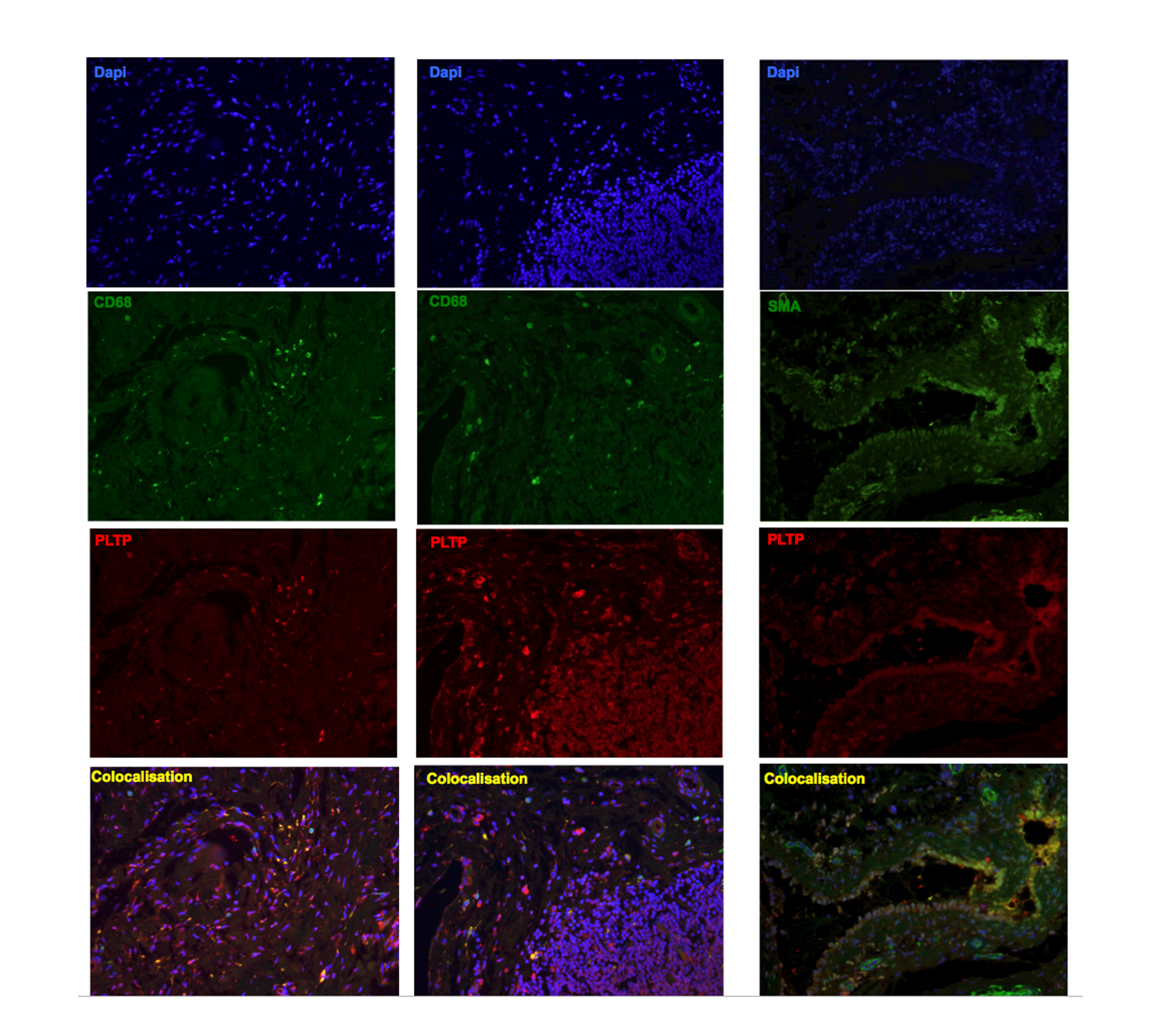

Supplement: S1 Fig — A) Double staining was performed to visualize PLTP localization. Synovial tissue sections from RA patients were stained for PLTP and macrophages (CD68+, left panel), or PLTP and RA-FLS (α-SMA+ cells) (n = 3). Fluorescence was analyzed at 20x magnification. Overlay is shown to visualize colocalization of PLTP in macrophages or in RA-FLS. Representative images obtained for immunohistological staining are shown. Original magnification: 20x. (TIF) [file pone.0193815.s001.tif]

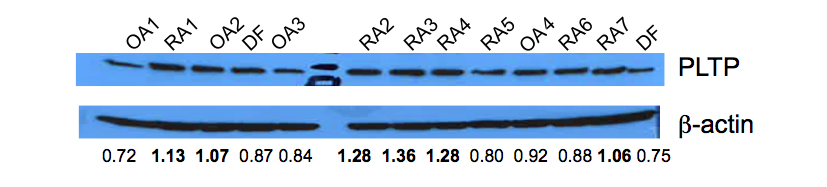

Supplement: S2 Fig — PLTP protein level in FLS was quantified by Western blot analysis, normalized using β-actin and then expressed as a ratio vs mean expression level in all FLS tested. A representative image is shown (RA: rheumatoid arthritis FLS, OA: osteoarthritis FLS; DF: normal dermal fibroblasts). (TIF) [file pone.0193815.s002.tif]

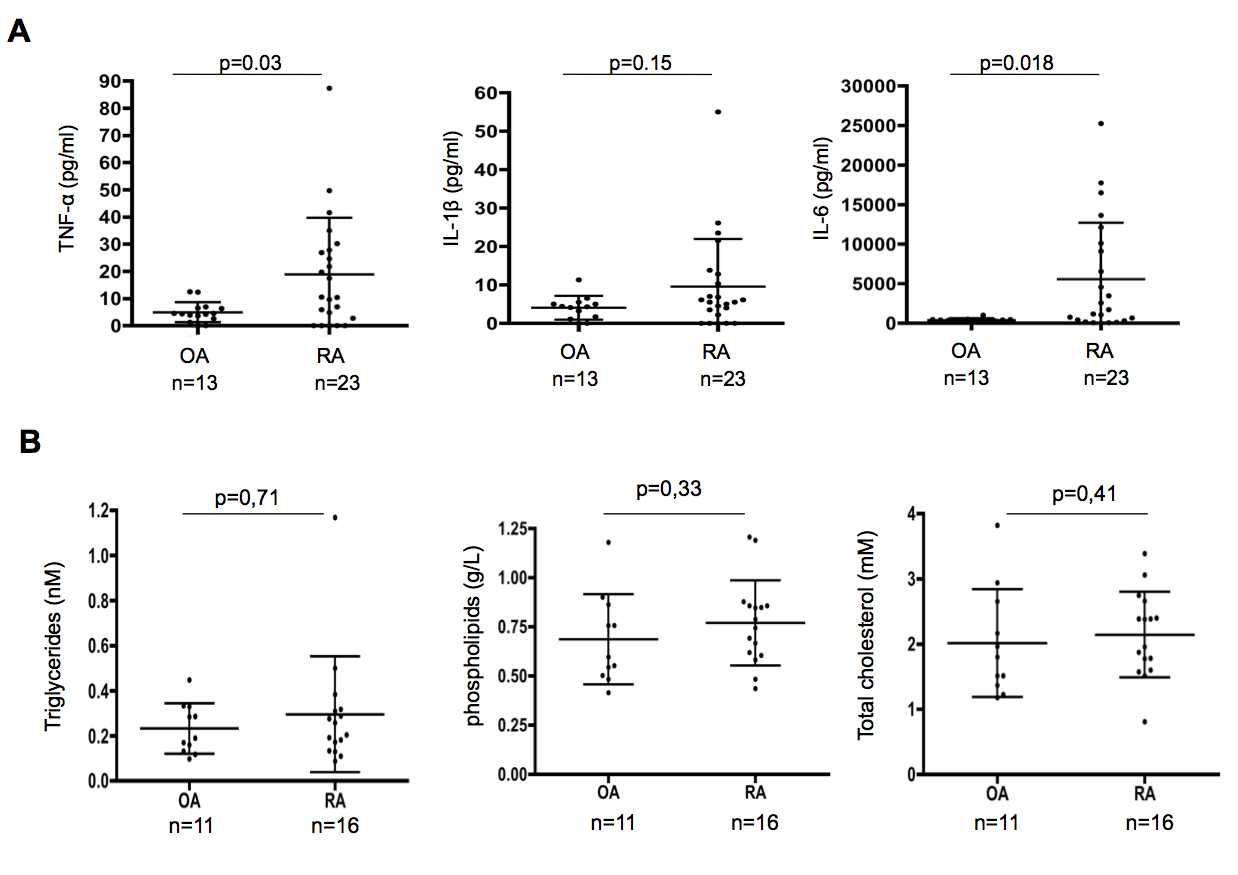

Supplement: S3 Fig — (A) Synovial fluids samples from patients were tested for IL-6, TNF-α and IL-1β concentrations using Milliplex MAP Human Cytokine/Chemokine Magnetic Bead Panel kit (Millipore, Billerica, MA). (B) Plasma lipids (total cholesterol, triglycerides and phospholipids) were assayed using commercially available kits on an Indiko Clinical chemistry analyzer (Thermo Fisher Scientific, Finland) according to the manufacturer’s instructions. Results are expressed as mean ± SD and statistical analysis performed using the Mann-Whitney test. (TIF) [file pone.0193815.s003.tif]

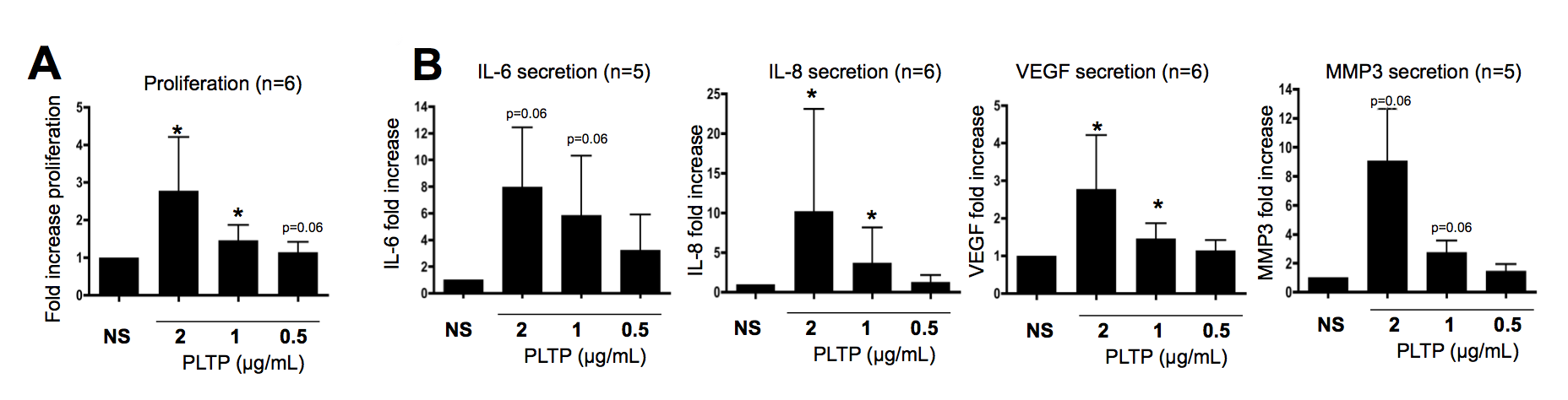

Supplement: S4 Fig — (A) FLS were stimulated for 48 hours with native PLTP at indicated concentrations and proliferation was assessed using [3H] thymidine incorporation during the last day of stimulation. Results are expressed as mean fold increase ± SD (n = 6). Statistical differences were assessed by Wilcoxon matched paired test. *p < 0.05 versus unstimulated conditions; NS: unstimulated (B) Effect of PLTP on FLS cytokine production. FLS were stimulated with native PLTP at indicated concentrations. Supernatants were then collected and assessed for cytokines (IL-6, IL-8, VEGF and MMP3) production by ELISA. Results are expressed as mean fold increase ± SD (n = 5 to 6). Statistical differences were assessed by Wilcoxon matched paired test. *p< 0.05 versus unstimulated conditions. (TIF) [file pone.0193815.s004.tif]

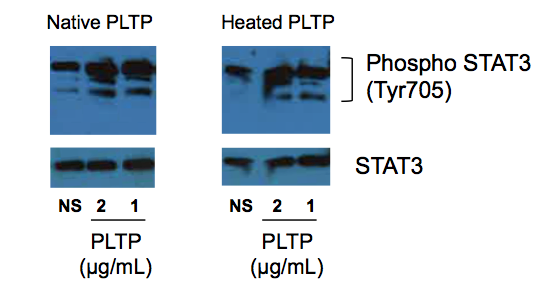

Supplement: S5 Fig — RA-FLS were stimulated for 24 hours with native PLTP (PLTP) or heat-inactivated PLTP (Heated PLTP) at the indicated concentrations. Cell lysates were analyzed by Western blot for phosphorylation of STAT3 (Tyr705). Band intensities were normalized to the corresponding band intensities for STAT3. (TIF) [file pone.0193815.s005.tif]
